# The Influence of Airborne Particulate Matter on the Risk of Gestational Diabetes Mellitus: A Large Retrospective Study in Chongqing, China

**DOI:** 10.3390/toxics12010019

**Published:** 2023-12-24

**Authors:** Xiaoling Zeng, Yu Zhan, Wei Zhou, Zhimei Qiu, Tong Wang, Qing Chen, Dandan Qu, Qiao Huang, Jia Cao, Niya Zhou

**Affiliations:** 1 Institute of Toxicology, Facutly of Military Preventive Medicine, Army Medical University (Third Military Medical University), Chongqing 400038, China; zxl19961104@163.com (X.Z.); wtyc112@163.com (T.W.); chenqingforward@gmail.com (Q.C.); 2 School of Public Health, China Medical University, Shenyang 110122, China; 3 Department of Environmental Science and Engineering, Sichuan University, Chengdu 610065, China; yzhan@scu.edu.cn (Y.Z.); 2020223055131@stu.scu.edu.cn (Z.Q.); 4 Department of Obstetrics and Gynecology, Chongqing Health Center for Women and Children (Women and Children’s Hospital of Chongqing Medical University), Chongqing 401147, China; dr.zhouwei@163.com (W.Z.); hq99202311@163.com (Q.H.); 5 Clinical Research Centre, Women and Children’s Hospital of Chongqing Medical University, Chongqing 401147, China; mangata1995@163.com; 6 Chongqing Research Centre for Prevention & Control of Maternal and Child Diseases and Public Health, Women and Children’s Hospital of Chongqing Medical University, Chongqing 401147, China

**Keywords:** airborne particulate matter, gestational diabetes mellitus, isolated post-load hyperglycemia, sensitive time windows, sensitive subtypes

## Abstract

Emerging research findings suggest that airborne particulate matter might be a risk factor for gestational diabetes mellitus (GDM). However, the concentration–response relationships and the susceptible time windows for different types of particulate matter may vary. In this retrospective analysis, we employ a novel robust approach to assess the crucial time windows regarding the prevalence of GDM and to distinguish the susceptibility of three GDM subtypes to air pollution exposure. This study included 16,303 pregnant women who received routine antenatal care in 2018–2021 at the Maternal and Child Health Hospital in Chongqing, China. In total, 2482 women (15.2%) were diagnosed with GDM. We assessed the individual daily average exposure to air pollution, including PM_2.5_, PM_10_, O_3_, NO_2_, SO_2_, and CO based on the volunteers’ addresses. We used high-accuracy gridded air pollution data generated by machine learning models to assess particulate matter per maternal exposure levels. We further analyzed the association of pre-pregnancy, early, and mid-pregnancy exposure to environmental pollutants using a generalized additive model (GAM) and distributed lag nonlinear models (DLNMs) to analyze the association between exposure at specific gestational weeks and the risk of GDM. We observed that, during the first trimester, per IQR increases for PM_10_ and PM_2.5_ exposure were associated with increased GDM risk (PM_10_: OR = 1.19, 95%CI: 1.07~1.33; PM_2.5_: OR = 1.32, 95%CI: 1.15~1.50) and isolated post-load hyperglycemia (GDM-IPH) risk (PM_10_: OR = 1.23, 95%CI: 1.09~1.39; PM_2.5_: OR = 1.38, 95%CI: 1.18~1.61). Second-trimester O_3_ exposure was positively correlated with the associated risk of GDM, while pre-pregnancy and first-trimester exposure was negatively associated with the risk of GDM-IPH. Exposure to SO_2_ in the second trimester was negatively associated with the risk of GDM-IPH. However, there were no observed associations between NO_2_ and CO exposure and the risk of GDM and its subgroups. Our results suggest that maternal exposure to particulate matter during early pregnancy and exposure to O_3_ in the second trimester might increase the risk of GDM, and GDM-IPH is the susceptible GDM subtype to airborne particulate matter exposure.

## 1. Introduction

Gestational diabetes mellitus (GDM) is a common metabolic disturbance of pregnancy. The condition increases the risk of complications for both diabetic mothers and infants, including maternal obesity [1,2,3], type 2 diabetes (T2DM), cardiovascular diseases [4,5], macrosomia, neonatal hypoglycemia, and long-term risk of obesity and cardiovascular diseases in offspring [6,7]. Over time, the global incidence of GDM has been on the rise. As ambient air pollution has become an important factor affecting human health, there are emerging studies showing that airborne particulate matter may contribute to GDM [8,9,10,11]. Both animal and population studies demonstrated that exposure to PM_2.5_ is positively linked to the risk of T2DM [12,13,14,15], affecting blood glucose through multiple pathways, including insulin resistance [16], endothelial dysfunction [17,18], and inflammatory responses [19,20]. Since pregnancy is a vulnerable period for women, there is increased interest in studying the effects of particulate matter on the onset of GDM and its further prevention in this particular population.

A precise exposure assessment method is crucial for estimating the effect of air pollutants on the risk of GDM. Most of the previous studies assessing maternal air pollution exposure levels were obtained from air monitoring stations. Observations at monitoring sites were inadequate to capture the spatial variation in air pollution at a fine scale, and thus assessing individual exposure with data from the nearest sites could cause substantial misclassification [21]. In recent years, studies have used a mixture of satellite simulation and monitoring data to estimate air pollution exposure. Machine learning models have been applied to predict the spatial and temporal distribution of atmospheric pollutants such as PM_2.5_, PM_10_, and O_3_. Machine learning algorithms may have higher predictive performance compared to traditional statistical models, such as general linear regression and kriging [22]. Random forest is a popular machine learning algorithm that makes statistical predictions by averaging over a collection of de-correlated classification or regression trees; it can handle nonlinear relationships and interaction effects [23]. Based on satellite data retrieval, ground-monitored nitrogen dioxide and carbon monoxide concentrations, and various geographic covariates, the use of spatiotemporal autocorrelation, random forest, and spatiotemporal kriging (RF-STK) models have also been proposed to predict daily ground-level nitrogen dioxide and carbon monoxide concentrations in different regions [24,25]. These data assimilation methods compensate for the high uncertainty of satellite retrieval and the low spatial coverage of ground-based detection, and effectively improve the spatial coverage and accuracy of pollutant exposure, providing more reliable information for environmental epidemiology studies and air quality management.

According to laboratory examinations, we can classify OGTT test results as normal glucose-tolerant (NGT) or as having isolated fasting hyperglycemia (GDM-IFH), isolated post-load hyperglycemia (GDM-IPH), or combined hyperglycemia (GDM-CH) [26]. More recently, emerging research has found that different subtypes of GDM may comprise different metabolic entities. Previous studies have found that fasting hyperglycemia (GDM-IFH) is closely associated with liver insulin sensitivity and subsequent liver glucose production, whereas post-load hyperglycemia (GDM-IPH) is closely linked with muscle insulin resistance [27,28]. Previous research has also indicated that GDM-IFH is strongly associated with adverse pregnancy outcomes, and these pregnant women have a greater need for insulin therapy and are less responsive to dietary lifestyle therapies [29,30]. However, to our knowledge, few studies have explored the impact of air pollution exposure on subclinical GDM groups during pregnancy. Therefore, it is of great importance to clarify the effects of ambient pollution exposure on GDM from a comprehensive viewpoint.

Chongqing is an industrial base in southwest China, where industry plays an important role in the development of its economy, and industrial pollution is the key cause of environmental pollution. Therefore, in this paper, we conduct a large retrospective study, which includes 16,303 participants, by employing our reliable air pollution assessment methods, aimed at (1) assessing the susceptible windows of air pollution exposure for GDM over the preconception period and first and second trimesters at weekly levels; and (2) distinguishing the specific air pollutants to which GDM and its subgroups are susceptible. This highly accurate individual pollutant exposure evaluation model and the two steps of statistical analysis strategies in our large sample study will provide high-level evidence for the association between air pollution exposure and the risk of GDM.

## 2. Methods and Materials

### 2.1. Study Population

This retrospective study included pregnant women who had their first prenatal care visit at the Chongqing Health Center for Women and Children, China, from January 2018 to June 2021. The recruiting criteria were pregnant women aged 18~49 years and who were long-term residents of Chongqing. The participants were excluded or ineligible for the study if they had T1DM or T2DM before pregnancy; had family members with diabetes; suffered from a serious psychiatric disorder; or did not complete the OGTT in the health center. The project proposal was approved by the Ethics Committee of the Chongqing Health Center for Women and Children.

**Figure 1:**
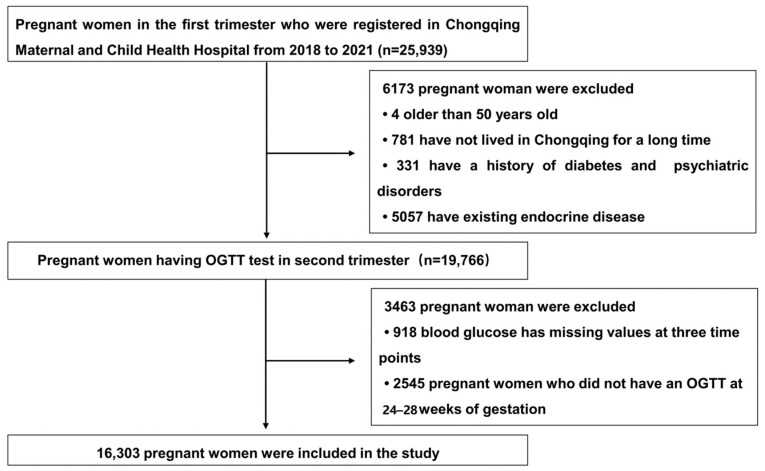
Flowchart of the study subjects’ recruitment.

A total of 25,939 volunteers were recruited and screened for participation in the study. Of these, 9636 pregnant women were ineligible or excluded from the final analysis, and the reasons included: being aged over 50 years (n = 4); not being a long-term resident of Chongqing (n = 781); having a history of diabetes, mental illness, and a family history of diabetes (n = 331); having an existing endocrine disease (excluding diabetes and other endocrine diseases, including thyroid, adrenal, and hypothalamic diseases; n = 5057); missing values for blood glucose at three time points (n = 918); and OGTT not performed at 24–28 weeks of gestation (n = 2545). Finally, 16,303 volunteers were included in the analysis. The flowchart of the recruitment of the volunteers included in this study is shown in Figure 1.

### 2.2. Glucose Tolerance Test and Diagnostic Criteria for GDM

According to the diagnostic criteria established by the International Consensus Group on Pregnancy with Diabetes (IADPSG) [31], pregnant women underwent the OGTT test at 24 to 28 weeks of gestation using the glucose oxidase assay (Hitachi 7600-110 fully automated biochemical analyzer, Tokyo, Japan). After fasting for a period ranging from 8 to 12 h the night before, venous blood was collected from the pregnant women in the next morning to measure the blood glucose. Then, 75 g of glucose was administered orally and blood was taken intravenously from the pregnant women again after 1 and 2 h. The diagnostic criteria for GDM were as follows: fasting glucose ≥ 5.1 mmol/L (92 mg/dL), 1 h post-glucose administration ≥ 10.0 mmol/L (180 mg/dL), or 2 h post-glucose administration ≥ 8.5 mmol/L (153 mg/dL). GDM can be diagnosed if any of the above conditions are met. Based on the results of the OGTT, pregnant women were classified as having isolated fasting hyperglycemia (GDM-IFH) if their fasting glucose was ≥5.1 mmol/L but their 1 h and 2 h post-load glucose levels were within the normal range, and as having isolated post-load hyperglycemia (GDM-IPH) if their 1 and 2 h post-load glucose levels were ≥10.0 mmol/L and ≥8.5 mmol/L, respectively. Pregnant women who exceeded the fasting and post-load glucose-restricted values were considered to have combined hyperglycemia (GDM-CH) [26].

### 2.3. Assessment of Individual Exposure to Air Pollutants and Meteorological Conditions

We assessed the individual daily average exposure to air pollution (including PM_2.5_, PM_10_, O_3_, NO_2_, SO_2_, and CO) and weather conditions (i.e., temperature and relative humidity) based on the long-term volunteers’ addresses and the spatially gridded datasets. A grid with a spatial resolution of 1×1 km^2^ was delineated for Chongqing. The daily average temperature and relative humidity observed at meteorological stations [32] were interpolated to all the grid cells using cokriging with elevation [33]. The data on daily air pollutant concentrations were obtained from the China National Environmental Monitoring Centre [34], which manages the air quality monitoring network across the nation. We developed hybrid machine learning models (i.e., random forest with spatiotemporal kriging) with air pollution observations and various predictor variables, such as satellite retrieval, weather conditions, and land uses [24,25], to predict the daily air pollutant concentrations for all the grid cells. As the key predictor, satellite retrieval mainly included the Multi-Angle Implementation of Atmospheric Correction (MAIAC) aerosol optical depth, the Ozone Monitoring Instrument (OMI) tropospheric vertical column density of NO_2_, the OMI vertical column density of SO_2_ in the planetary boundary layer, and the Measurements of Pollution in the Troposphere (MOPITT) CO retrieval [35,36,37,38].

### 2.4. Statistical Analysis

The categorical variables are represented by frequency (n) or percentage (%), and the chi-squared test was used to compare binary variables and unordered multi-category variables between groups. The correlation coefficients (r) of air pollutants and meteorological factors were analyzed using Spearman’s correlation to evaluate collinearity in the regression analysis.

We then analyzed the association of pre-pregnancy, early, and mid-pregnancy exposure to environmental pollutants with GDM, GDM-IFH, GDM-IPH, and GDM-CH using a generalized additive model (GAM). In the GAM, the response variable can have any distribution in the exponential family [39]. The GAM model can identify nonlinear associations among variables. This model maximizes the predictive quality of the responses by fitting a more flexible model to the data. ORs with 95% CIs were reported for per IQR increases in NO_2_, O_3_, PM_10_, PM_2.5_, SO_2_, and CO concentrations during each exposure window. We also established a two-pollutant model to evaluate whether the risk of GDM from the studied pollutants changed after controlling for other pollutants unless the Spearman’s correlation coefficient of the two pollutants was greater than 0.6 [40]. We performed stratified analyses according to the OGTT sampling time (cold and warm seasons), age (<35 and ≥35 years old), and BMI (<24 and ≥24 kg/cm^2^). Likelihood ratio tests were used to calculate interaction *p*-values.

In addition, we used distributed lag nonlinear models (DLNMs) to analyze the association between exposure at specific gestational weeks and the risk of GDM [41]. We analyzed the exposure and lag effects for three trimesters (preconception: weeks −12 to −1; first trimester: weeks 1 to 12; and second trimester: weeks 13 to 24). Since all pregnant women in this study had OGTT at 24 to 28 weeks and the diagnosis of GDM was made immediately based on the test results, the 24th week after the last menstrual period was used as the cutoff time. ORs and 95% CIs were calculated for each increase in IQR (study period of 2018–2021) for different pollutants. When constructing the regression model of environmental pollution exposure with GDM, the maternal age, first-trimester BMI, tobacco, alcoholism, gravidity, parity, macrosomia secretion, assisted reproduction, multiple pregnancies, and sampling season of OGTT (spring, summer, autumn, and winter) were controlled. All of the above covariates were categorical variables. The natural cubic spline function was used to control meteorological factors, such as temperature and relative humidity (RH), and the degrees of freedom of the temperature and RH in each exposure time window were selected based on the minimum Akaike information criterion (AIC). A small amount of missing data were filled using the predicted mean matching method in Multiple Imputation (MI) [42].

The baseline data of the study subjects were analyzed using SPSS 25.0. A distributed lag nonlinear model analysis was performed using the “dlnm”, “mgcv”, and “splines” packages of R 4.1.2. *p* < 0.05 was considered statistically significant. For GDM and blood glucose, Bonferroni correction *p* < 0.006 (0.05/8) was used to assess statistical significance [43].

## 3. Results

### 3.1. Description of the Baseline Information

The demographics of the participants are presented in Table 1. From January 2018 to June 2021, 16,303 pregnant women were included in the final analysis. Among them, there were 2482 cases (15.2%) with GDM, including 214 (1.3%) with GDM-IFH, 1692 (10.4%) with GDM-IPH, and 284 (1.7%) with GDM-CH. Compared with non-GDM pregnant women, advanced age and overweight or obese mothers were more common in the GDM group and subgroups. More pregnant women in the GDM group and subgroups exhibited gravidity ≥ 3, parity ≥ 1, and had undergone assisted reproduction. The proportion of twin pregnancies was greater in non-GDM pregnant women. Among all pregnant women in our study, the average fasting blood glucose levels were 4.43 ± 0.39 mmol/L, the average 1 h post-glucose level was 7.73 ± 1.74 mmol/L, and the average 2 h post-glucose level was 6.65 ± 1.41 mmol/L (Table 2).

### 3.2. Air Pollution Exposure

The average levels of maternal exposure to NO_2_, O_3_, PM_10_, PM_2.5_, SO_2_, and CO over the preconception period were 40.96 ± 8.66, 42.03 ± 18.44, 60.41 ± 16.50, 37.27 ± 13.46, 8.63 ± 1.48 μg/m^3^, and 0.86 ± 0.13 mg/m^3^, respectively, similar to those in the first and second trimesters (Table 2). The average temperature and relative humidity were also similar across different gestation periods, and in the preconception period, they were 17.92 °C and 80.34%, respectively. The Spearman’s correlation analysis of air pollutants and meteorological factors is shown in Figure S1 in the Supplementary Materials. Correlations among NO_2_, O_3_, PM_10_, PM_2.5_, CO, SO_2_, temperature, and relative humidity weekly levels ranged from −0.77 to 0.94. O_3_ and meteorological factors were negatively correlated with other air pollutants.

### 3.3. Association of Air Pollution Exposure with GDM and Its Subgroups

**Figure 2:**
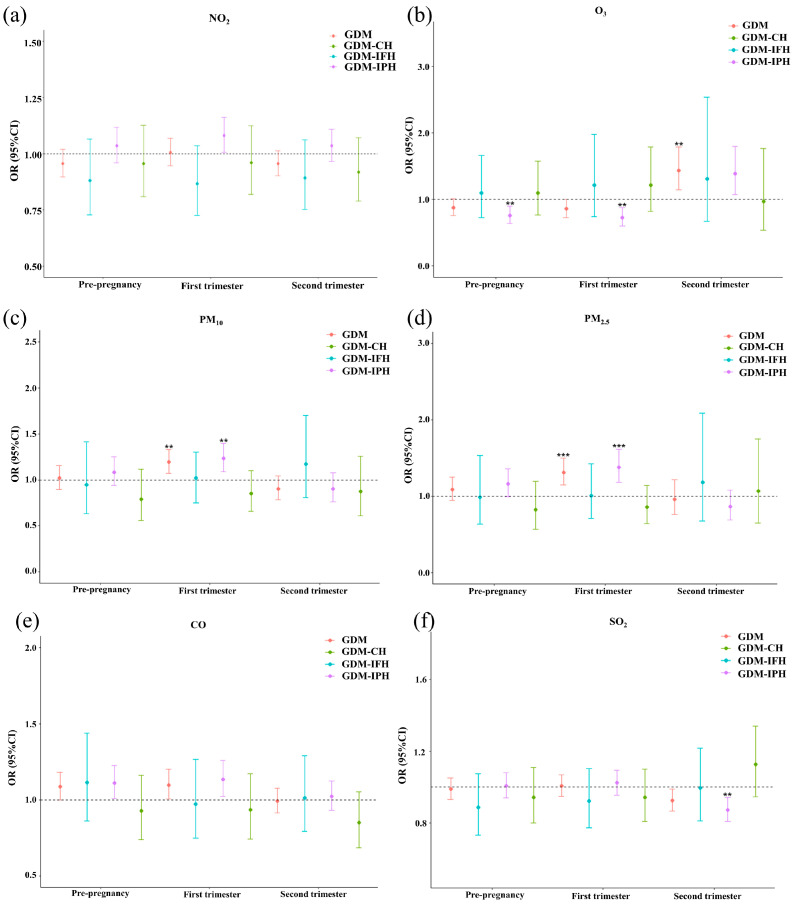
Adjusted odds ratios (ORs) and 95% confidence intervals (CIs) for air pollution exposure (per IQR) and risk of GDM, GDM-IFH, GDM-IPH, and GDM-CH in single-pollutant models, 2018–2021. (**a**) Effect of NO_2_ exposure during different trimesters on the risk of GDM and various subgroups. (**b**) Effect of O_3_ exposure during different trimesters on the risk of GDM and various subgroups. (**c**) Effect of PM_10_ exposure during different trimesters on the risk of GDM and various subgroups. (**d**) Effect ofPM_2.5_ exposure during different trimesters on the risk of GDM and various subgroups. (**e**) Effect of CO exposure during different trimesters on the risk of GDM and various subgroups. (**f**) Effect of SO_2_ exposure during different trimesters on the risk of GDM and various subgroups. Bonferroni corrections with significance (*p* < 0.006), ** *p* < 0.006, and *** *p* < 0.001. Abbreviations: GDM, gestational diabetes mellitus; GDM-IFH, GDM with isolated fasting hyperglycemia; GDM-IPH, GDM with isolated post-load hyperglycemia; GDM-CH, GDM with combined hyperglycemia; NO_2_, nitrogen dioxide; O_3_, ozone; PM_10_, inhalable particulate matter; PM_2.5_, fine particulate matter; CO, carbon monoxide; SO_2_, sulfur dioxide. Model adjusted for maternal age, first-trimester BMI, gravidity, parity, tobacco, alcohol, folic acid, assisted reproduction, macrosomia, multiple pregnancies, season of OGTT, temperature, and relative humidity.

Figure 2 shows that the effects of per IQR increase in exposure to PM_10_ and PM_2.5_ during the first trimester were associated with increased GDM (PM_10_: OR = 1.19, 95%CI: 1.07~1.33; PM_2.5_: OR = 1.32, 95%CI: 1.15~1.50) and GDM-IPH risks (PM_10_: OR = 1.23, 95%CI: 1.09~1.39; PM_2.5_: OR = 1.38, 95%CI: 1.18~1.61). Per IQR O_3_ exposure during the second trimester increased the associated risks of GDM by 43% (95%CI: 15%~79%), while preconception and first-trimester exposure was negatively associated with GDM-IPH risks. Each IQR increase in SO_2_ in the second trimester was negatively associated with the risk of GDM-IPH. However, there were no observed associations between NO_2_ and CO exposure and the risk of GDM, GDM-IFH, GDM-IPH, and GDM-CH. We also constructed a two-pollutant model, and similar associations were observed between air pollutants and the risk of GDM and its subgroups (see Tables S1–S4 in the Supplementary Materials).

### 3.4. Association between Air Pollutant Exposure and GDM in Specific Gestational Weeks

**Figure 3:**
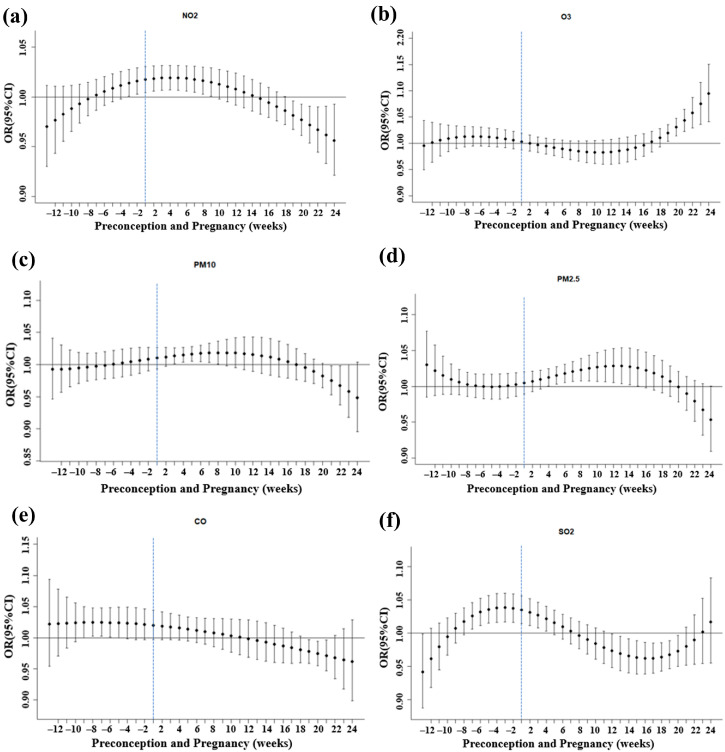
Adjusted odds ratios (ORs) and 95% confidence intervals (CIs) for the association of week-specific air pollution exposure (per IQR) with GDM risk in pregnant women, 2018–2021. (**a**) Effect of NO_2_ exposure at different gestational weeks on GDM. (**b**) Effect of O_3_ exposure at different gestational weeks on GDM. (**c**) Effect of PM_10_ exposure at different gestational weeks on GDM. (**d**) Effect of PM_2.5_ exposure at different gestational weeks on GDM. (**e**) Effect of CO exposure at different gestational weeks on GDM. (**f**) Effect of SO_2_ exposure at different gestational weeks on GDM. Preconception: weeks −12 to −1; first trimester: weeks 1 to 12; and second trimester: weeks 13 to 24. Abbreviations: GDM, gestational diabetes mellitus; NO_2_, nitrogen dioxide; O_3_, ozone; PM_10_, inhalable particulate matter; PM_2.5_, fine particulate matter; CO, carbon monoxide; SO_2_, sulfur dioxide. Model adjusted for maternal age, first-trimester BMI, gravidity, parity, tobacco, alcohol, folic acid, assisted reproduction, macrosomia, multiple pregnancies, season of OGTT, temperature, and relative humidity.

The multivariable-adjusted associations of GDM with week-specific air pollutant exposure during the preconception period and first and second trimesters are shown in Figure 3. A positive correlation between per IQR increase in NO_2_ and GDM was observed from −2 to 9 weeks, with the strongest association from 2 to 5 weeks (OR = 1.02, 95%CI: 1.01~1.03). The critical time window for O_3_ exposure was 19 to 24 weeks, with the strongest effects observed at week 24 (OR = 1.09, 95%CI: 1.04~1.15). PM_10_ and PM_2.5_ increases per IQR were positively correlated with GDM risk at 3 to 8 and 4 to 15 weeks, with the strongest association in week 7 (OR = 1.02, 95%CI: 1.00~1.03) and week 12 (OR = 1.03, 95%CI: 1.01~1.05), respectively. CO exposure from −8 to −5 weeks was associated with the risk of GDM, with the strongest association at week −7 (OR = 1.02, 95%CI: 1.00~1.05). Exposure to SO_2_ from −6 to 4 weeks was positively associated with the risk of GDM, with the strongest effect at week −2 (OR = 1.04, 95%CI: 1.02~1.06).

### 3.5. Stratified Analysis and Interaction Tests

The subgroup analysis results show a greater association of air pollutants with GDM and GDM-IPH during the warm season and in normal or lean women, with no significant differences in the age groups (see Figures S2–S4 in the Supplementary Materials). The results of the interaction analysis suggest that the seasons and BMI had potential modification effects on the association of environmental pollution exposure and GDM.

## 4. Discussion

This is the first large-population-based study to assess air pollutant exposure and GDM risk in southwest China, and one of the few studies to evaluate the relationship between air pollution and the risk of GDM in various subgroups. Our research found that maternal exposure to PM_10_ and PM_2.5_ was positively correlated with the risk of GDM and GDM-IPH, and the susceptible exposure windows for PM_10_ and PM_2.5_ were observed at weeks 3 to 8 and 4 to 15, with the strongest associations found at weeks 7 and 12, when the risk of GDM increased by 2.0% (95% CI: 0.0%~3.0%) and 3.0% (95% CI: 1.0%~5.0%) for each increase in IQR for PM_10_ and PM_2.5_, respectively. A susceptibility exposure window for O_3_ was observed at weeks 19 to 24 of gestation, with the strongest association found at 24 weeks of pregnancy, with a 9.0% (95% CI: 4.0%~15%) increased risk of GDM per IQR increase in O_3_.

Most previous investigations have applied land-use regression (LUR) models based on data from monitoring networks, and these data were all based on the census mesh block level or location of the hospital. However, the use of such relatively extensive exposure data may lead to erroneous estimates. Moreover, most monitoring sites are clustered in urban areas, and a lack or paucity of sites are available in suburban or rural areas. In this study, we used a mixture of satellite simulation and monitoring data to estimate individual air pollution levels based on every mother’s residential address. In addition, we used the gridded air pollution data generated by machine learning models to assess individual exposure levels, which improved the exposure classification and reduced the bias in the exposure–effect analyses. Our previous studies used high-accuracy gridded air pollution data generated by machine learning models to assess exposure levels and demonstrated that different fractions of PAHs in fine particulate matter probably have different effects on male reproductive health [44]. The machine learning models that are capable of handling complicated nonlinear interactions showed a decent performance in the cross-validation. Gridded datasets have been used for exposure assessments in previous studies [45,46,47]. Compared to the nearest-site matching method for exposure assessment, machine learning models provided more accurate air pollution data by fusing site observations with various environmental factors, such as land-use types and satellite retrieval [48,49]. Machine learning models demonstrated a superior performance in reconstructing the spatiotemporal distributions of air pollutants, which laid a solid basis for exposure–effect analyses [45,47,50]. The precision and robustness of these evaluation methods have been well demonstrated in our previous studies [51,52].

Our study confirmed the significant positive correlation between air pollutant PM_10_ and PM_2.5_ exposure and GDM. Although previous epidemiological evidence supports the air pollution effect on GDM risk, these results remain heterogeneous [53,54,55,56,57]. These inconsistent results can be attributed to ethnic variations, regional differences, and different time periods for air pollution assessment. Many studies have evaluated the window of sensitivity to air pollution, which can help to determine the potential pathways of pathogenesis and guide care during pregnancy. There are three strategies for estimating the window of susceptibility to air pollution exposure during pregnancy and GDM, including by specific trimester, by month, and by week. Previous studies have concentrated on the first and second trimesters. For example, a meta-analysis that included 22,253,277 participants found that exposure to ambient pollutants during early pregnancy was connected to pregnancy complications [58]. According to a cohort study conducted in Foshan, China, 12,842 maternal exposures to PM_10_ and PM_2.5_ in early and middle pregnancy were associated with the risk of GDM [57]. However, emerging research evidence suggests that the pre-pregnancy period is also a critical exposure window for ambient pollution exposure that affects GDM [59]. Other studies have shown that a relatively broad, specific three-month window of exposure may mask the true effects of contaminants because biological changes do not exactly follow the three-month interval [60]. Numerous studies have observed the correlation between the risk of GDM and maternal exposure to environmental pollutants [54,55,56,57,58,61,62,63,64]. Wilson [60] suggested that the use of a relatively broad, specific three-month exposure window may mask the true effects of contaminants because biological changes do not exactly follow the three-month interval. Physiological changes throughout pregnancy usually occur on a weekly basis and include endocrine, cardiovascular, respiratory, and water balance [65,66]. We used more refined weekly exposure data for further analysis, and the DLNM results show that weeks 3 to 8 and 4 to 15 are sensitive time windows for PM_10_ and PM_2.5_ exposure with the effects peaking at weeks 7 and 12, respectively,. Our study found that particulate matter exposure was associated with early pregnancy GDM. This adds new evidence to the study of environmental particulate matter exposure and the GDM risk sensitivity window and provides important guidance for reducing environmental particulate matter exposure in early pregnancy to control the occurrence of pregnancy complications related to air pollution.

Different from particulate matter, exposure to O_3_ in mid-pregnancy was also positively correlated with the risk of GDM. It was found that the susceptible exposure window was 19 to 24 weeks using DLNMs to explore the week-specific association, with the maximum effect being reached at 24 weeks. O_3_ concentrations at ambient temperature have highly oxidizing properties and can cause damage to the organism, but the underlying mechanisms remain unclear. Wagner JG [67] found that short-term repeated O_3_ exposure in mice induced a pulmonary inflammatory response, which was correlated with the degree of insulin resistance and hyperglycemia. Zhong JX [68] found that the continuous exposure of genetically susceptible diabetic mice to O_3_ for 13 working days promoted insulin resistance and that exposure to O_3_ can increase oxidative stress and the inflammatory response of adipose tissue. Insulin resistance is considered to be an important cause of GDM [69,70], and O_3_ exposure may increase the risk of GDM by promoting insulin resistance.

Our study also explored the risk of ambient pollutant exposure and GDM subtypes to provide effective and individualized treatment strategies. We observed that PM_10_ and PM_2.5_ exposure in early pregnancy and O_3_ exposure in mid-pregnancy were associated with an increased risk of GDM-IPH, but not significantly correlated with the risk of GDM-IFH. This suggests that maternal exposure to air pollutants during pregnancy may increase the incidence of GDM by influencing postprandial glucose abnormalities. Recent evidence suggests that abnormal fasting and abnormal post-load hyperglycemia reflect different metabolic processes and that mothers with isolated post-load hyperglycemia tend to have unfavorable metabolic profiles compared to those with isolated fasting hyperglycemia [26]. Clinical studies have found that the sites of insulin resistance occurring for impaired postprandial glucose and impaired fasting glucose are different. Patients with impaired postprandial glucose show significant muscle insulin resistance, but those with impaired fasting glucose exhibit more pronounced hepatic insulin resistance [71]. Haberzettl et al.’s [11] study indicated that, in mice on a high-fat diet, exposure to concentrated environmental fine particulate matter enhances adipose tissue inflammation and systemic glucose intolerance. Another animal study revealed that ozone exposure in rats promotes the development of diabetes by activating the JNK pathway to impair insulin signaling in muscles [72]. These potential mechanisms may explain the differential association we observed between ambient pollutant exposure and various subtypes of GDM, suggesting that ambient pollutant exposure may ultimately increase the risk of GDM by promoting muscle insulin resistance, leading to postprandial hyperglycemia.

Previous studies have evaluated the relationship between exposure to NO_2_, CO, and SO_2_ and GDM during specific trimesters, and the results indicated that SO_2_ exposure in the preconception period and early pregnancy was significantly correlated with the risk of GDM, particularly from 4 to 10 weeks of gestation [53,73]. Liu [74] found that CO exposure in early pregnancy was significantly associated with GDM. Another study observed the connection between NO_2_ exposure and GDM in a different model and found that the preconception period was the critical window, while the association in early pregnancy was not statistically significant [55]. However, the study showed no significant positive correlation between NO_2_, CO, and SO_2_ exposure and GDM in single and co-pollution models. In addition, we used DLNMs to ascertain the susceptibility window between gaseous pollutant exposure and GDM risk at the weekly level. By applying DLNMs, we observed that the preconception period and the first trimester are windows of susceptibility for different gaseous pollutants (NO_2_: weeks −2~9; CO: weeks −8~−5; SO_2_: weeks −6~4), with peak associations observed at weeks −7 to −2 and 2 to 5, respectively.

In addition, our study also found that, after stratifying by cold and warm seasons according to the OGTT trial season, pregnant women in the warm season were at a greater risk for GDM from PM_10_ and PM_2.5_ exposure in early pregnancy and O_3_ exposure in mid-pregnancy. PM_10_ and PM_2.5_ exposure in early pregnancy interacted with the OGTT season. The risk of GDM was higher in the warm season, possibly reflecting the effect of ambient temperature on glucose metabolism. Previous studies have reported that GDM development is influenced by the season, with an increased risk of GDM in the warm season. And temperature was negatively correlated with fasting glucose and positively correlated with post-load glucose [75]. Retnakaran [76] found that an elevated ambient temperature may lead to maternal β-cell dysfunction, thereby increasing the risk of GDM. The stratified analysis of BMI revealed an effect modification of BMI and air pollution exposure with GDM, with a positive association between air pollution exposure and GDM in pregnant women with BMI < 24. Numerous studies have shown that obesity leads to mild chronic systemic inflammation and oxidative stress that persist in the body [77,78]. Therefore, pregnant women with BMI < 24 may be more sensitive to inflammation and oxidative stress attributable to environmental exposures compared to overweight or obese pregnant women. No significant interaction of age with air pollution exposure was observed in this study. However, the results of the stratified analysis must be interpreted with caution, and type 1 errors (false positives) may be introduced in multiple trials. Further in-depth studies are needed regarding the possible effects of ambient temperature and BMI on glucose metabolism and their potential biological mechanisms.

In this retrospective study in Chongqing, China, the possibility of selection bias was decreased by recruiting pregnant women who came to the Chongqing Health Center for Women and Children for regular prenatal visits and obtained OGTT results. All investigators involved in this study were formally provided with uniform training to ensure the quality of information. A highly refined, spatiotemporally resolved exposure model was used to assess individual air pollution exposure concentrations, and a two-step statistical analysis strategy was used to explore the sensitive time window of exposure from shallow to deep, which is more robust and reliable than the results of previous studies. This study also has several limitations. Firstly, we estimated individual air pollution exposure levels using the home address of the pregnant women and did not consider their individual activity patterns during pregnancy, including commuting, time spent working in different environments, and time spent outdoors. Secondly, pregnant women usually undergo OGTT screening for GDM in the late second trimester; therefore, we can only assume that testing occurred between 24 and 28 weeks of gestation based on the IADPSG criteria and recommendations, which may lead to a potential misclassification of the exposure time estimates. Finally, this study was a single-center retrospective study with a sample from a single hospital; all baseline information was obtained through the medical center’s electronic record access system, and some covariate data were missing from the records. Therefore, prospective, multicenter, and larger studies must be conducted in the future for support and validation.

## 5. Conclusions

Our findings indicate that exposure to PM_10_ and PM_2.5_ in the first trimester and O_3_ in the second trimester is associated with an increased risk of GDM and GDM-IPH, providing strong evidence for an association between airborne particulate matter and the risk of GDM and glucose metabolism disorders. In addition, the sensitive time window of weekly air pollutant exposure levels for GDM risk was analyzed. Our findings are instructive for the prevention and treatment of GDM from an environmental perspective, and more studies are needed to confirm our findings and explore potential mechanisms.

## Figures and Tables

**Table 1:** The basic characteristics of the study population, 2018–2021.

Categories	Total	Non-GDM	GDM	GDM-IFH	GDM-IPH	GDM-CH
	n (%)					
	16,303	13,821 (84.8)	2482 (15.2)	214 (1.3)	1692 (10.4)	284 (1.7)
Age (years)						
<25	436	408 (3.0)	28 (1.1)	4 (1.9)	20 (1.2)	2 (0.7)
25–30	4015	3666 (26.5)	349 (14.1)	46 (21.5)	229 (13.5)	24 (8.5)
31–35	6613	5701 (41.2)	912 (36.7)	79 (36.9)	625 (36.9)	94 (33.1)
≥35	5239	4046 (29.3)	1193 (48.1)	85 (39.7)	818 (48.3)	164 (57.7)
BMI (kg/cm^2^)						
<18.5	4287	3984 (28.8)	303 (12.2)	24 (11.2)	250 (14.8)	6 (2.1)
18.5–24.9	9354	7900 (57.2)	1454 (58.6)	114 (53.3)	1029 (60.8)	138 (48.6)
25.0–29.9	2273	1643 (11.9)	630 (25.4)	67 (31.3)	362 (21.4)	122 (43.0)
≥30	389	294 (2.1)	95 (3.8)	9 (4.2)	51 (3.0)	18 (6.3)
Gravidity						
1	5097	4445 (32.2)	652 (26.3)	57 (26.6)	468 (27.7)	65 (22.9)
2	4367	3735 (27.0)	632 (25.5)	51 (23.8)	430 (25.4)	69 (24.3)
≥3	6839	5641 (40.8)	1198 (48.3)	106 (49.5)	794 (46.9)	150 (52.8)
Parity						
0	9851	8479 (61.3)	1372 (55.3)	114 (53.3)	949 (56.1)	158 (55.6)
≥1	6452	5342 (38.7)	1110 (44.7)	100 (46.7)	743 (43.9)	126 (44.4)
Tobacco						
Yes	490	411 (3.0)	79 (3.2)	3 (1.4)	51 (3.0)	16 (5.6)
No	15,813	13,410 (97.0)	2403 (96.8)	211 (98.6)	1641 (97.0)	268 (94.4)
Alcoholism						
Yes	2484	2116 (15.3)	368 (14.8)	39 (18.2)	234 (13.8)	48 (16.9)
No	13,819	11,705 (84.7)	2114 (85.2)	175 (81.8)	1458 (86.2)	236 (83.1)
Folic acid						
Yes	15,725	13,333 (96.5)	2392 (96.4)	210 (98.1)	1628 (96.2)	271 (95.4)
No	578	488 (3.5)	90 (3.6)	4 (1.9)	64 (3.8)	13 (4.6)
Multiple pregnancy						
Yes	892	690 (5.0)	202 (8.1)	17 (7.9)	140 (8.3)	27 (9.5)
No	15,411	13,131 (95.0)	2280 (91.9)	197 (92.1)	1552 (91.7)	257 (90.5)
Macrosomia						
Yes	31	27 (0.2)	4 (0.2)	0 (0)	2 (0.1)	2 (0.7)
No	16,272	13,794 (99.8)	2478 (99.8)	214 (100)	1690 (99.9)	282 (99.3)
ART						
Yes	1537	1190 (8.6)	347 (14.0)	26 (12.1)	242 (14.3)	42 (14.8)
No	14,766	12,631 (91.4)	2135 (86.0)	188 (87.9)	1450 (85.7)	242 (85.2)
Sampling Season						
Spring	4515	3806 (27.5)	709 (28.6)	57 (26.6)	489 (28.9)	84 (29.6)
Summer	4512	3820 (27.6)	692 (27.9)	44 (20.6)	331 (19.6)	72 (25.4)
Autumn	3872	3301 (23.9)	571 (23.0)	43 (20.1)	394 (23.3)	57 (20.1)
Winter	3404	2894 (20.9)	510 (20.5)	70 (32.7)	478 (28.3)	71 (25.0)

Abbreviations: GDM, gestational diabetes mellitus; BMI, body mass index; ART, assisted reproductive technology; GDM-IFH, GDM with isolated fasting hyperglycemia; GDM-IPH, GDM with isolated post-load hyperglycemia; GDM-CH, GDM with combined hyperglycemia.

**Table 2:** Descriptive statistics of air pollution exposure and blood glucose levels, 2018–2021.

	Total				Non-GDM				GDM				*p*
	Mean ± SD	P_25_	P_50_	P_75_	Mean ± SD	P_25_	P_50_	P_75_	Mean ± SD	P_25_	P_50_	P_75_	
Preconception													
NO_2_ (μg/m^3^)	40.96 ± 8.66	35.80	42.07	46.89	40.97 ± 8.66	35.81	42.02	46.93	40.90 ± 8.69	35.73	42.20	46.68	0.848
O_3_ (μg/m^3^)	42.03 ± 18.44	23.53	43.71	58.01	42.19 ± 18.43	23.63	44.14	58.12	41.17 ± 18.49	23.26	41.46	57.23	0.020
PM_10_ (μg/m^3^)	60.41 ± 16.50	47.05	57.55	71.62	60.37 ± 16.52	47.02	57.55	71.52	60.65 ± 16.42	47.17	57.51	72.20	0.383
PM_2.5_ (μg/m^3^)	37.27 ± 13.46	26.21	33.41	47.30	37.21 ± 13.48	26.19	33.34	47.14	37.60 ± 13.35	26.37	33.74	47.88	0.119
CO (mg/m^3^)	0.86 ± 0.13	0.76	0.85	0.95	0.86 ± 0.13	0.76	0.85	0.95	0.87 ± 0.13	0.77	0.86	0.96	0.008
SO_2_ (μg/m^3^)	8.63 ± 1.48	7.64	8.53	9.38	8.62 ± 1.48	7.63	8.51	9.36	8.69 ± 1.49	7.69	8.58	9.48	0.015
Temperature	17.92 ± 6.52	11.91	18.02	23.78	17.95 ± 6.53	11.94	18.12	23.78	17.75 ± 6.50	11.79	17.63	23.79	0.153
RH	80.34 ± 3.91	76.89	79.97	83.98	80.29 ± 3.91	76.83	79.89	83.90	80.62 ± 3.89	77.15	80.37	84.38	<0.001
First trimester													
NO_2_ (μg/m^3^)	40.67 ± 8.61	35.68	41.89	46.59	40.60 ± 8.60	35.57	41.81	46.57	41.02 ± 8.67	36.09	42.29	46.74	0.016
O_3_ (μg/m^3^)	41.80 ±18.89	22.78	43.38	58.19	42.02 ± 18.85	23.01	43.88	58.31	40.54 ± 19.02	21.75	40.60	57.38	<0.001
PM_10_ (μg/m^3^)	60.60 ± 16.07	47.18	58.89	71.99	60.28 ± 16.02	46.91	58.51	71.53	62.37 ± 16.23	48.67	60.84	74.00	<0.001
PM_2.5_ (μg/m^3^)	37.86 ± 13.45	26.29	34.78	49.24	37.60 ± 13.41	26.13	34.23	48.83	39.33 ± 13.60	27.21	37.21	50.69	<0.001
CO (mg/m^3^)	0.85 ± 0.12	0.77	0.85	0.94	0.85 ± 0.12	0.77	0.85	0.93	0.86 ± 0.12	0.78	0.86	0.96	<0.001
SO_2_ (μg/m^3^)	8.52 ± 1.39	7.61	8.47	9.28	8.50 ± 1.39	7.59	8.45	9.28	8.59 ± 1.39	7.70	8.53	9.29	0.009
Temperature	17.47 ± 6.78	10.75	17.09	23.78	17.58 ± 6.78	10.89	17.28	24.02	16.82 ± 6.73	10.19	16.22	22.95	<0.001
RH	80.07 ± 3.67	76.95	79.65	82.99	80.06 ± 3.67	76.94	79.62	82.95	80.12 ± 3.65	77.04	79.74	83.24	0.355
Second trimester													
NO_2_ (μg/m^3^)	40.27 ± 8.36	35.88	41.44	46.12	40.33 ± 8.37	35.97	41.54	46.17	39.96 ± 8.31	35.44	41.05	45.82	0.010
O_3_ (μg/m^3^)	43.49± 18.04	26.93	45.80	58.43	43.29 ± 18.07	26.45	45.55	58.32	44.61 ± 17.84	29.42	47.57	58.98	0.002
PM_10_ (μg/m^3^)	57.93 ± 13.78	47.08	56.49	67.73	57.94 ± 13.77	47.11	56.49	67.73	57.85 ± 13.84	46.78	56.60	67.79	0.832
PM_2.5_ (μg/m^3^)	35.72 ± 11.77	26.14	32.96	44.80	35.72 ± 11.79	26.12	32.92	44.81	35.69 ± 11.68	26.15	33.08	44.73	0.982
CO (mg/m^3^)	0.84 ± 0.12	0.76	0.83	0.91	0.84 ± 0.12	0.76	0.83	0.92	0.83 ± 0.12	0.75	0.83	0.91	0.204
SO_2_ (μg/m^3^)	8.32 ± 1.15	7.58	8.29	9.05	8.33 ± 1.16	7.58	8.29	9.05	8.31 ± 1.15	7.58	8.31	9.06	0.820
Temperature	18.14 ± 6.28	12.22	18.26	23.73	18.12 ± 6.28	12.22	18.26	23.70	18.21 ± 6.31	12.16	18.27	23.90	0.473
RH	80.05 ± 3.63	76.93	79.58	82.95	80.11 ± 3.63	76.97	79.70	83.01	79.68 ± 3.61	76.75	79.01	82.59	<0.001
OGTT glucose levels (mmol/L)													
Fasting glucose	4.43 ± 0.39	4.20	4.40	4.59	4.36 ± 0.30	4.09	4.40	4.59	4.85 ± 0.53	4.50	4.80	5.20	<0.001
1 h post-glucose	7.73 ± 1.74	6.50	7.70	8.80	7.29 ± 1.37	6.30	7.40	8.30	10.18 ± 1.54	9.40	10.10	11.00	<0.001
2 h post-glucose	6.65 ± 1.41	5.70	6.50	7.40	6.28 ± 1.03	5.60	6.30	7.00	8.68 ± 1.51	7.70	8.69	9.50	<0.001

Abbreviations: GDM, gestational diabetes mellitus; SD, standard deviation; RH, relative humidity; NO_2_, nitrogen dioxide; O_3_, ozone; PM_10_, inhalable particulate matter; PM_2.5_, fine particulate matter; CO, carbon monoxide; SO_2_, sulfur dioxide. The rank sum test was used to compare the levels of air pollutants and blood glucose in the GDM and non-GDM groups.

## Data Availability

The data presented in this study are available upon request from the corresponding authors. Due to the protection of volunteer privacy, the data were not made public.

## Supplementary Materials

The following supporting information can be downloaded at: https://www.mdpi.com/article/10.3390/toxics12010019/s1, Table S1: Adjusted odds ratios (ORs) and 95% confidence intervals (CIs) for air pollution exposure and the risk of GDM in co-pollutant models, 2018–2021. Table S2: Adjusted odds ratios (ORs) and 95% confidence intervals (CIs) for air pollution exposure and the risk of GDM-IFH in co-pollutant models, 2018–2021. Table S3: Adjusted odds ratios (ORs) and 95% confidence intervals (CIs) for air pollution exposure and the risk of GDM-IPH in co-pollutant models, 2018–2021. Table S4: Adjusted odds ratios (ORs) and 95% confidence intervals (CIs) for air pollution exposure and the risk of GDM-CH in co-pollutant models, 2018–2021. Figure S1: Spearman’s correlation analysis of specific gestational pollutants and meteorological factors. Figure S2: Modification for potential effects of air pollution exposure in association with GDM subgroups according to cold and warm seasons. Figure S3: Modification for the potential effects of air pollution exposure in association with GDM subgroups according to maternal age. Figure S4: Modification for the potential effects of air pollution exposure in association with GDM subgroups according to BMI in the first trimester.
