# Supplementary material for: The Influence of Airborne Particulate Matter on the Risk of Gestational Diabetes Mellitus: A Large Retrospective Study in Chongqing, China
Source: Toxics. 2023 Dec 24;12(1):19. doi: 10.3390/toxics12010019 (PMC10818620; doi:10.3390/toxics12010019)
Supplement: Supplementary file 1 [file toxics-12-00019-s001.zip › toxics-2743860-supplementary.pdf]

Table S1. Adjusted odds ratios (ORs) and 95% confidence intervals (CIs) for air pollution exposure and the risk of GDM in co-pollutant models, 2018-2021.

		Pre-pregnancy		First trimester		Second trimester	
	**Adjusting for**	**OR**	**95% (CI)**	**OR**	**95% (CI)**	**OR**	**95% (CI)**
**NO_2**							
	+O_3	0.92	(0.86~0.99)	0.97	(0.89~1.04)	1.04	(0.96~1.13)
	+PM_{10}	0.95	(0.89~1.02)	0.96	(0.89~1.02)	0.98	(0.91~1.05)
	+$PM_{2.5}$	0.95	(0.89~1.01)	0.97	(0.91~1.03)	0.97	(0.91~1.03)
	+CO	0.94	(0.88~1.01)	0.99	(0.92~1.05)	0.97	(0.91~1.03)
	+SO_2	0.96	(0.90~1.02)	1.01	(0.94~1.07)	0.97	(0.91~1.03)
**O_3**							
	+NO_2	0.82	(0.70~0.96)	0.80	(0.65~0.98)	1.58	(1.16~2.14) **
	+PM_{10}	0.85	(0.72~1.00)	0.99	(0.81~1.20)	1.41	(1.11~1.78) **
	+$PM_{2.5}$	0.88	(0.74~1.05)	1.04	(0.85~1.26)	1.43	(1.14~1.78) **
	+CO	0.92	(0.78~1.09)	0.90	(0.75~1.06)	1.44	(1.15~1.80) **
	+SO_2	0.87	(0.76~1.01)	0.86	(0.73~1.01)	1.39	(1.11~1.74) **
**PM_{10}**							
	+NO_2	1.05	(0.91~1.20)	1.24	(1.10~1.39) **	0.94	(0.79~1.11)
	+O_3	0.95	(0.81~1.10)	1.20	(1.05~1.36) **	0.97	(0.83~1.13)
	+CO	0.95	(0.82~1.09)	1.17	(1.04~1.31)	0.90	(0.78~1.05)
	+SO_2	1.04	(0.90~1.19)	1.24	(1.10~1.40) **	0.93	(0.80~1.08)
**$PM_{2.5}$**							
	+NO_2	1.10	(0.96~1.27)	1.34	(1.17~1.55) ***	1.00	(0.78~1.26)

	$+O_3$	1.02	(0.86~1.20)	1.34	(1.14~1.57) ***	0.99	(0.79~1.25)
	$+SO_2$	1.11	(0.96~1.29)	1.38	(1.19~1.61) ***	0.98	(0.78~1.24)
**CO**							
	$+NO_2$	1.10	(1.01~1.20)	1.11	(1.01~1.21)	1.00	(0.92~1.09)
	$+O_3$	1.06	(0.97~1.17)	1.09	(0.99~1.20)	1.01	(0.93~1.10)
	$+PM_{10}$	1.11	(1.01~1.22)	1.05	(0.95~1.16)	1.00	(0.92~1.08)
	$+SO_2$	1.09	(1.01~1.19)	1.10	(1.01~1.20)	0.99	(0.91~1.07)
**SO_2**							
	$+NO_2$	0.99	(0.93~1.05)	1.01	(0.95~1.07)	0.93	(0.87~0.99)
	$+O_3$	0.99	(0.93~1.05)	1.00	(0.94~1.06)	0.94	(0.88~1.00)
	$+PM_{10}$	0.98	(0.92~1.05)	0.95	(0.89~1.02)	0.93	(0.87~1.00)
	$+PM_{2.5}$	0.97	(0.91~1.04)	0.95	(0.89~1.01)	0.93	(0.87~0.99)
	+CO	0.98	(0.92~1.04)	1.00	(0.94~1.06)	0.92	(0.87~0.99)

Bonferroni corrections with significance ($p < 0.006$), ** $p<0.006$, and *** $p<0.001$.

Abbreviations: GDM, gestational diabetes mellitus; NO_2, nitrogen dioxide; O_3, ozone; PM_{10}, inhalable particulate matter; $PM_{2.5}$, fine particulate matter; CO, carbon monoxide; SO_2, sulfur dioxide.

Model adjusted for maternal age, first-trimester BMI, gravidity, parity, tobacco, alcohol, folic acid, assisted reproduction, macrosomia, multiple pregnancies, the season of OGTT, temperature, and relative humidity.

Table S2. Adjusted odds ratios (ORs) and 95% confidence intervals (CIs) for air pollution exposure and the risk of GDM-IFH in co-pollutant models, 2018-

2021.

	Adjusting for	Pre-pregnancy OR	Pre-pregnancy 95% (CI)	First trimester OR	First trimester 95% (CI)	Second trimester OR	Second trimester 95% (CI)
**NO_2**							
	$+O_3$	0.87	(0.70~1.07)	0.86	(0.70~1.07)	0.89	(0.71~1.12)
	$+PM_{10}$	0.85	(0.70~1.04)	0.84	(0.70~1.02)	0.85	(0.70~1.03)
	$+PM_{2.5}$	0.87	(0.72~1.06)	0.86	(0.72~1.03)	0.88	(0.74~1.05)
	+CO	0.86	(0.71~1.05)	0.86	(0.71~1.04)	0.89	(0.74~1.06)
	$+SO_2$	0.88	(0.73~1.07)	0.87	(0.73~1.04)	0.89	(0.75~1.06)
**O_3**							
	$+NO_2$	0.90	(0.56~1.44)	0.98	(0.55~1.74)	0.98	(0.41~2.35)
	$+PM_{10}$	1.09	(0.69~1.71)	1.39	(0.76~2.55)	1.42	(0.71~2.84)
	$+PM_{2.5}$	1.11	(0.70~1.75)	1.38	(0.75~2.54)	1.35	(0.69~2.65)
	+CO	1.22	(0.76~1.93)	1.24	(0.72~2.15)	1.34	(0.68~2.66)
	$+SO_2$	1.08	(0.71~1.65)	1.17	(0.71~1.93)	1.31	(0.67~2.55)
**PM_{10}**							
	$+NO_2$	1.09	(0.71~1.66)	1.14	(0.82~1.57)	1.38	(0.88~2.15)
	$+O_3$	0.98	(0.63~1.51)	1.15	(0.79~1.66)	1.26	(0.82~1.93)
	+CO	0.87	(0.56~1.35)	1.04	(0.73~1.48)	1.19	(0.78~1.82)
	$+SO_2$	1.07	(0.69~1.67)	1.13	(0.79~1.61)	1.20	(0.79~1.84)
**$PM_{2.5}$**							
	$+NO_2$	1.05	(0.67~1.64)	1.08	(0.76~1.53)	1.26	(0.72~2.21)
	$+O_3$	1.03	(0.64~1.67)	1.16	(0.75~1.78)	1.23	(0.70~2.16)

	$+SO_2$	1.10	(0.68~1.77)	1.11	(0.75~1.64)	1.20	(0.67~2.12)
**CO**							
	$+NO_2$	1.16	(0.90~1.51)	1.04	(0.79~1.36)	1.05	(0.82~1.33)
	$+O_3$	1.16	(0.88~1.54)	1.03	(0.77~1.37)	1.04	(0.81~1.33)
	$+PM_{10}$	1.15	(0.87~1.52)	0.96	(0.71~1.30)	1.00	(0.78~1.28)
	$+SO_2$	1.15	(0.88~1.48)	1.00	(0.77~1.30)	1.01	(0.80~1.29)
**SO_2**							
	$+NO_2$	0.89	(0.73~1.07)	0.94	(0.78~1.12)	0.99	(0.79~1.22)
	$+O_3$	0.89	(0.73~1.07)	0.93	(0.78~1.12)	1.00	(0.80~1.23)
	$+PM_{10}$	0.88	(0.71~1.08)	0.89	(0.72~1.09)	0.98	(0.77~1.21)
	$+PM_{2.5}$	0.88	(0.72~1.07)	0.90	(0.74~1.10)	0.98	(0.78~1.21)
	+CO	0.88	(0.72~1.06)	0.92	(0.77~1.11)	0.99	(0.79~1.22)

Bonferroni corrections with significance ($p < 0.006$), ** $p<0.006$, and *** $p<0.001$.

Abbreviations: GDM-IFH, GDM with isolated fasting hyperglycaemia; NO_2, nitrogen dioxide; O_3, ozone; PM_{10}, inhalable particulate matter; $PM_{2.5}$, fine particulate matter; CO, carbon monoxide; SO_2, sulfur dioxide.

Model adjusted for maternal age, first-trimester BMI, gravidity, parity, tobacco, alcohol, folic acid, assisted reproduction, macrosomia, multiple pregnancies, the season of OGTT, temperature, and relative humidity.

Table S3. Adjusted odds ratios (ORs) and 95% confidence intervals (CIs) for air pollution exposure and the risk of GDM-IPH in co-pollutant models, 2018-2021.

	**Pre-pregnancy**		**First trimester**		**Second trimester**	

	Adjusting for	OR	95% (CI)	OR	95% (CI)	OR	95% (CI)
**NO_2**							
	+O_3	0.98	(0.90~1.06)	1.02	(0.93~1.11)	1.17	(1.07~1.29) **
	+PM_{10}	1.02	(0.94~1.11)	1.03	(0.95~1.12)	1.08	(0.99~1.17)
	+$PM_{2.5}$	1.02	(0.95~1.10)	1.04	(0.96~1.12)	1.04	(0.97~1.12)
	+CO	1.02	(0.94~1.10)	1.06	(0.98~1.14)	1.03	(0.96~1.11)
	+SO_2	1.04	(0.96~1.12)	1.08	(1.00~1.16)	1.03	(0.96~1.11)
**O_3**							
	+NO_2	0.75	(0.62~0.90) **	0.76	(0.61~0.95)	2.05	(1.44~2.91) ***
	+PM_{10}	0.74	(0.61~0.89) **	0.85	(0.67~1.09)	1.35	(1.03~1.77)
	+$PM_{2.5}$	0.76	(0.62~0.93)	0.89	(0.69~1.13)	1.38	(1.06~1.79)
	+CO	0.79	(0.65~0.95)	0.77	(0.63~0.96)	1.41	(1.08~1.83)
	+SO_2	0.76	(0.64~0.90) **	0.73	(0.60~0.89) **	1.32	(1.02~1.72)
**PM_{10}**							
	+NO_2	1.07	(0.91~1.24)	1.21	(1.05~1.38)	0.82	(0.67~1.01)
	+O_3	0.95	(0.80~1.12)	1.16	(1.00~1.35)	0.96	(0.80~1.15)
	+CO	1.00	(0.85~1.18)	1.19	(1.04~1.37)	0.89	(0.75~1.07)
	+SO_2	1.10	(0.93~1.29)	1.29	(1.12~1.49) **	0.95	(0.80~1.13)
**$PM_{2.5}$**							
	+NO_2	1.15	(0.99~1.35)	1.35	(1.15~1.59) ***	0.84	(0.67~1.06)
	+O_3	1.01	(0.84~1.22)	1.31	(1.09~1.57) **	0.89	(0.71~1.11)
	+SO_2	1.18	(1.00~1.40)	1.46	(1.22~1.74) ***	0.90	(0.72~1.13)

**CO**							
	$+NO_2$	1.11	(1.00~1.22)	1.11	(1.00~1.24)	1.02	(0.92~1.12)
	$+O_3$	1.04	(0.93~1.16)	1.09	(0.97~1.21)	1.04	(0.95~1.15)
	$+PM_{10}$	1.11	(1.00~1.24)	1.07	(0.95~1.20)	1.03	(0.94~1.14)
	$+SO_2$	1.12	(1.01~1.23)	1.13	(1.02~1.26)	1.01	(0.92~1.11)
**SO_2**							
	$+NO_2$	1.01	(0.94~1.08)	1.01	(0.95~1.09)	0.87	(0.81~0.94) **
	$+O_3$	1.00	(0.93~1.07)	1.00	(0.94~1.08)	0.88	(0.81~0.95) **
	$+PM_{10}$	0.99	(0.91~1.07)	0.95	(0.88~1.03)	0.88	(0.81~0.95) **
	$+PM_{2.5}$	0.98	(0.91~1.06)	0.95	(0.88~1.03)	0.87	(0.81~0.94) **
	+CO	0.99	(0.93~1.07)	1.01	(0.94~1.08)	0.87	(0.81~0.94) **

Bonferroni corrections with significance ($p < 0.006$), ** $p < 0.006$, and *** $p < 0.001$.

Abbreviations: GDM-IPH, GDM with isolated post-load hyperglycemia; NO_2, nitrogen dioxide; O_3, ozone; PM_{10}, inhalable particulate matter; $PM_{2.5}$, fine particulate matter; CO, carbon monoxide; SO_2, sulfur dioxide.

Model adjusted for maternal age, first-trimester BMI, gravidity, parity, tobacco, alcohol, folic acid, assisted reproduction, macrosomia, multiple pregnancies, the season of OGTT, temperature, and relative humidity.

Table S4. Adjusted odds ratios (ORs) and 95% confidence intervals (CIs) for air pollution exposure and the risk of GDM-CH in co-pollutant models, 2018-2021.

	Adjusting for	Pre-pregnancy		First trimester		Second trimester	
		**OR**	**95% (CI)**	**OR**	**95% (CI)**	**OR**	**95% (CI)**
**NO_2**							
	$+O_3$	0.97	(0.80~1.17)	1.00	(0.83~1.21)	0.86	(0.70~1.05)
	$+PM_{10}$	1.00	(0.83~1.20)	1.00	(0.84~1.20)	0.93	(0.78~1.10)
	$+PM_{2.5}$	0.97	(0.82~1.15)	0.98	(0.83~1.16)	0.91	(0.78~1.07)
	+CO	0.97	(0.82~1.14)	0.97	(0.82~1.15)	0.94	(0.80~1.09)
	$+SO_2$	0.96	(0.81~1.13)	0.97	(0.82~1.14)	0.92	(0.79~1.07)
**O_3**							
	$+NO_2$	1.06	(0.71~1.60)	1.22	(0.76~1.94)	0.67	(0.31~1.45)
	$+PM_{10}$	0.98	(0.66~1.47)	1.06	(0.64~1.76)	0.90	(0.48~1.69)
	$+PM_{2.5}$	1.02	(0.68~1.52)	1.11	(0.67~1.83)	0.98	(0.54~1.80)
	+CO	1.05	(0.70~1.58)	1.19	(0.77~1.85)	0.90	(0.49~1.63)
	$+SO_2$	1.09	(0.76~1.56)	1.18	(0.79~1.77)	1.04	(0.57~1.89)
**PM_{10}**							
	$+NO_2$	0.79	(0.55~1.15)	0.85	(0.64~1.13)	0.95	(0.63~1.42)
	$+O_3$	0.78	(0.53~1.15)	0.87	(0.63~1.21)	0.86	(0.59~1.25)
	+CO	0.79	(0.53~1.17)	0.84	(0.62~1.14)	0.93	(0.65~1.35)
	$+SO_2$	0.80	(0.55~1.17)	0.86	(0.64~1.16)	0.82	(0.57~1.19)
**$PM_{2.5}$**							
	$+NO_2$	0.84	(0.57~1.22)	0.86	(0.64~1.17)	1.12	(0.68~1.83)

	$+O_3$	0.83	(0.55~1.26)	0.90	(0.62~1.30)	1.06	(0.65~1.75)
	$+SO_2$	0.85	(0.57~1.27)	0.88	(0.63~1.22)	1.02	(0.62~1.68)
**CO**							
	$+NO_2$	0.94	(0.74~1.18)	0.95	(0.75~1.20)	0.87	(0.70~1.08)
	$+O_3$	0.94	(0.73~1.22)	0.98	(0.76~1.26)	0.85	(0.68~1.05)
	$+PM_{10}$	1.00	(0.78~1.29)	1.02	(0.78~1.33)	0.86	(0.69~1.07)
	$+SO_2$	0.94	(0.75~1.18)	0.95	(0.75~1.20)	0.86	(0.69~1.07)
**SO_2**							
	$+NO_2$	0.94	(0.80~1.11)	0.95	(0.81~1.11)	1.13	(0.95~1.34)
	$+O_3$	0.94	(0.80~1.11)	0.96	(0.82~1.12)	1.13	(0.95~1.35)
	$+PM_{10}$	0.99	(0.82~1.18)	0.99	(0.83~1.19)	1.15	(0.96~1.37)
	$+PM_{2.5}$	0.97	(0.81~1.15)	0.97	(0.82~1.16)	1.13	(0.94~1.34)
	+CO	0.95	(0.80~1.12)	0.95	(0.81~1.11)	1.12	(0.94~1.33)

Bonferroni corrections with significance ($p < 0.006$), ** $p < 0.006$, and *** $p < 0.001$.

Abbreviations: GDM-CH, GDM with combined hyperglycemia; NO_2, nitrogen dioxide; O_3, ozone; PM_{10}, inhalable particulate matter; $PM_{2.5}$, fine particulate matter; CO, carbon monoxide; SO_2, sulfur dioxide.

Model adjusted for maternal age, first-trimester BMI, gravidity, parity, tobacco, alcohol, folic acid, assisted reproduction, macrosomia, multiple pregnancies, the season of OGTT, temperature, and relative humidity.

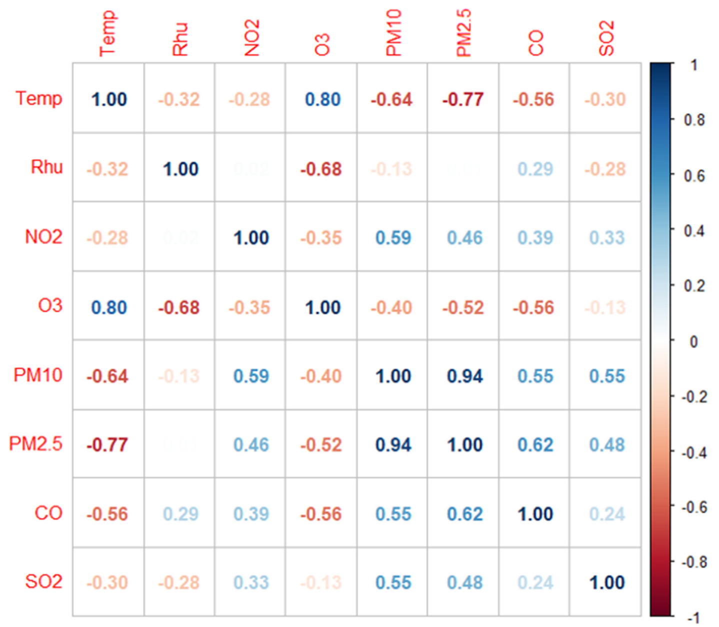

Figure S1. Spearman's correlation analysis of specific gestational pollutants and meteorological factors.

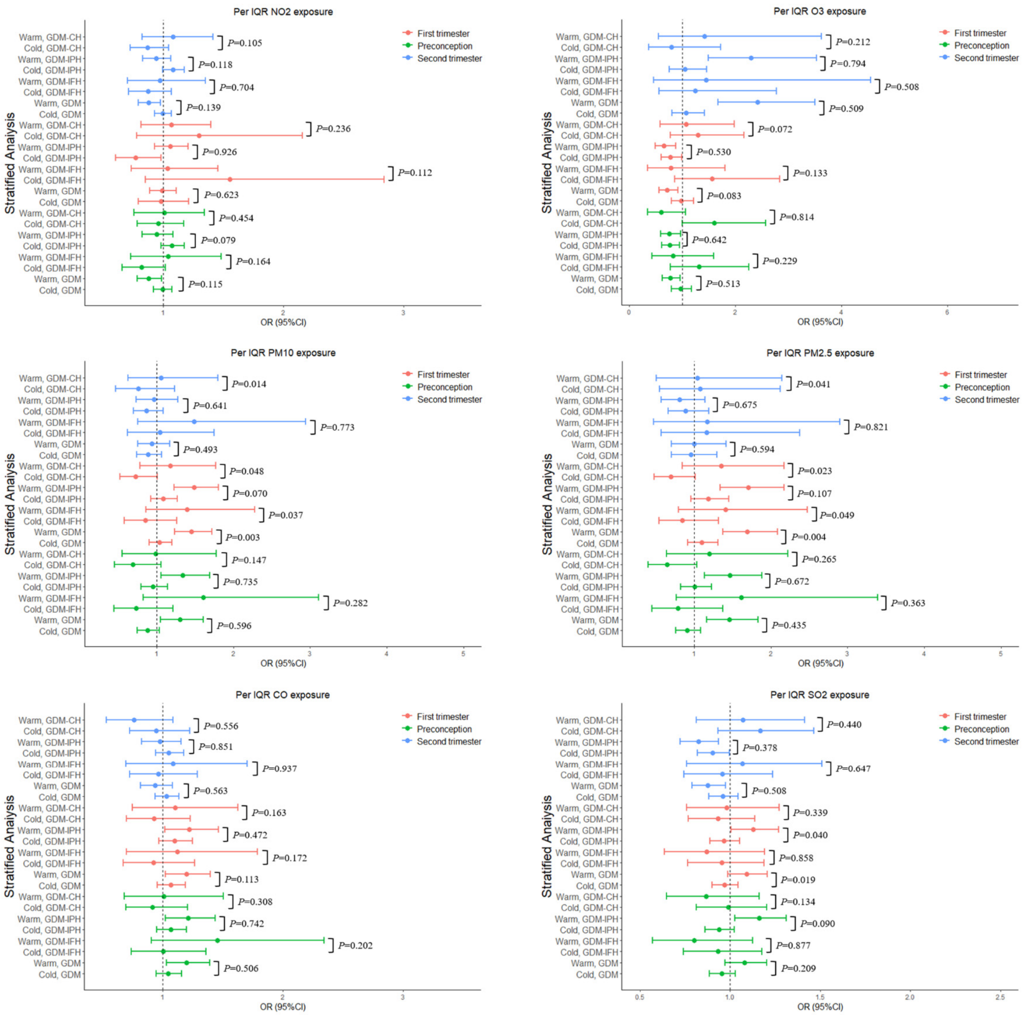

Figure S2. Modification for the potential effects of air pollution exposure in association with GDM subgroups according to cold and warm seasons. *P*-value is

shown for interaction. Abbreviations: GDM, gestational diabetes mellitus; GDM-IFH, GDM with isolated fasting hyperglycemia; GDM-IPH, GDM with isolated post-load hyperglycemia; GDM-CH, GDM with combined hyperglycemia; NO_2, nitrogen dioxide; O_3, ozone; PM_{10}, inhalable particulate matter; $PM_{2.5}$, fine particulate matter; CO, carbon monoxide; SO_2, sulfur dioxide; IQR, interquartile range.

Model adjusted for maternal age, first-trimester BMI, gravidity, parity, tobacco, alcohol, folic acid, assisted reproduction, macrosomia, multiple pregnancies, temperature, and relative humidity.

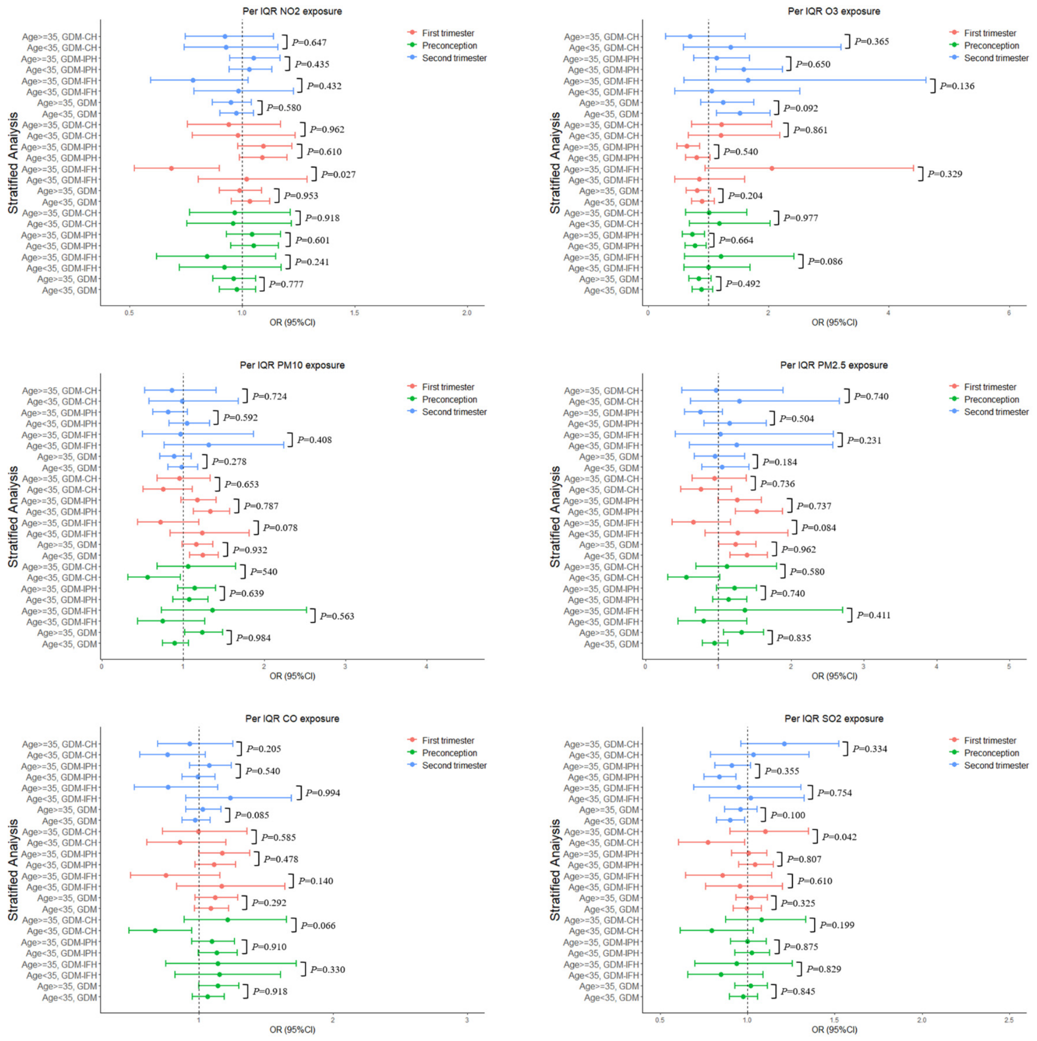
Per IQR NO2 exposure
Per IQR O3 exposure
Per IQR PM10 exposure
Per IQR PM2.5 exposure
Per IQR CO exposure
Per IQR SO2 exposure
First trimester
Preconception
Second trimester
Stratified Analysis
OR (95%CI)
Age>=35, GDM-CH
Age<35, GDM-CH
Age>=35, GDM-IPH
Age<35, GDM-IPH
Age>=35, GDM-IFH
Age<35, GDM-IFH
Age>=35, GDM
Age<35, GDM
P=0.647
P=0.435
P=0.432
P=0.580
P=0.962
P=0.610
P=0.027
P=0.953
P=0.918
P=0.601
P=0.241
P=0.777
P=0.365
P=0.650
P=0.136
P=0.092
P=0.861
P=0.540
P=0.329
P=0.204
P=0.977
P=0.664
P=0.086
P=0.492
P=0.724
P=0.592
P=0.408
P=0.278
P=0.653
P=0.787
P=0.078
P=0.932
P=540
P=0.639
P=0.563
P=0.984
P=0.740
P=0.504
P=0.231
P=0.184
P=0.736
P=0.737
P=0.084
P=0.962
P=0.580
P=0.740
P=0.411
P=0.835
P=0.205
P=0.540
P=0.994
P=0.085
P=0.585
P=0.478
P=0.140
P=0.292
P=0.066
P=0.910
P=0.330
P=0.918
P=0.334
P=0.355
P=0.754
P=0.100
P=0.042
P=0.807
P=0.610
P=0.325
P=0.199
P=0.875
P=0.829
P=0.845

Figure S3. Modification for the potential effects of air pollution exposure in association with GDM subgroups according to maternal age. *P*-value is shown for interaction. Abbreviations: GDM, gestational diabetes mellitus; GDM-IFH, GDM with isolated fasting hyperglycemia; GDM-IPH, GDM with isolated post-load hyperglycemia; GDM-CH, GDM with combined hyperglycemia; NO_2, nitrogen dioxide; O_3, ozone; PM_{10}, inhalable particulate matter; $PM_{2.5}$, fine particulate matter; CO, carbon monoxide; SO_2, sulfur dioxide; IQR, interquartile range.

Model adjusted for first-trimester BMI, gravidity, parity, tobacco, alcohol, folic acid, assisted reproduction, macrosomia, multiple pregnancies, the season of OGTT，temperature, and relative humidity.

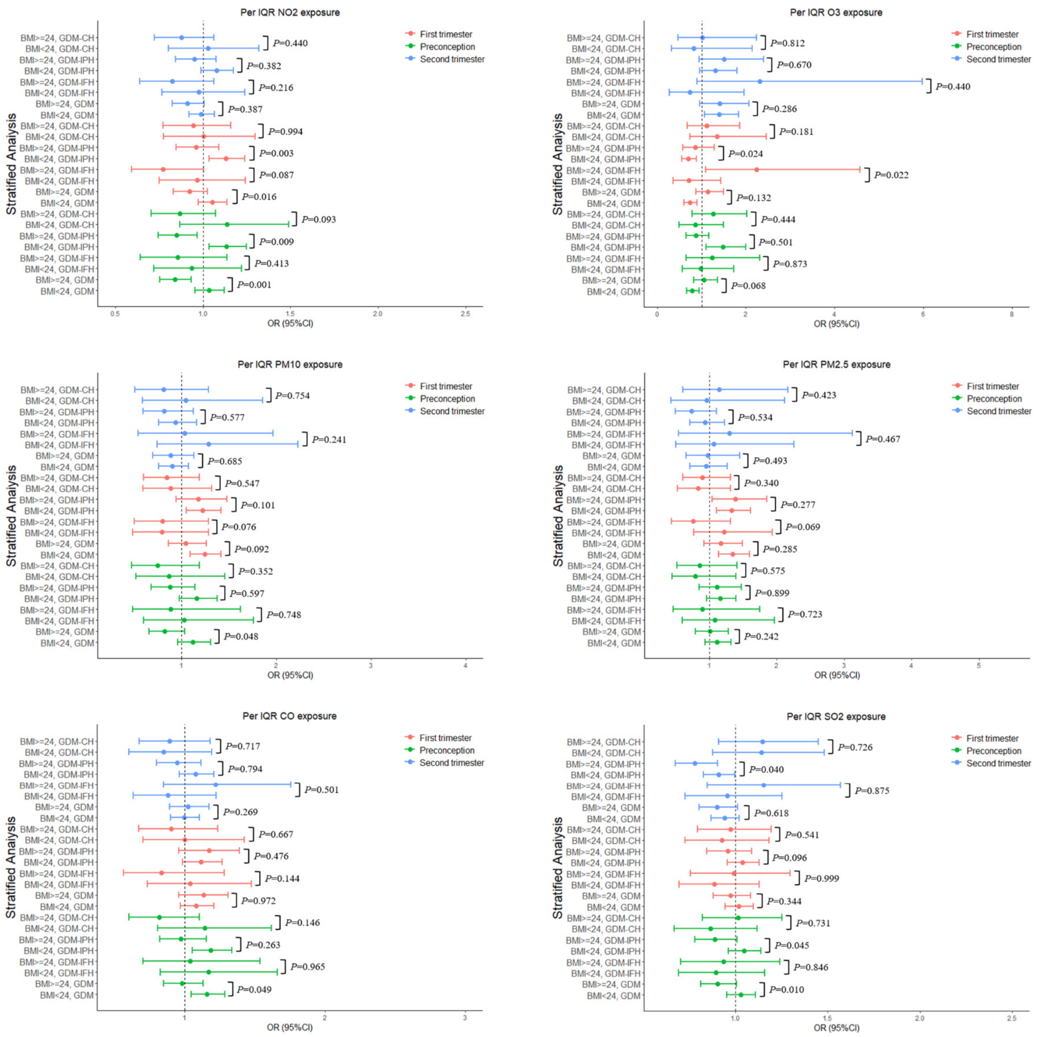
Per IQR NO2 exposure
Stratified Analysis
BMI>=24, GDM-CH
BMI<24, GDM-CH
BMI>=24, GDM-IPH
BMI<24, GDM-IPH
BMI>=24, GDM-IFH
BMI<24, GDM-IFH
BMI>=24, GDM
BMI<24, GDM
P=0.440
P=0.382
P=0.216
P=0.387
P=0.994
P=0.003
P=0.087
P=0.016
P=0.093
P=0.009
P=0.413
P=0.001
OR (95%CI)
First trimester
Preconception
Second trimester
Per IQR O3 exposure
P=0.812
P=0.670
P=0.440
P=0.286
P=0.181
P=0.024
P=0.022
P=0.132
P=0.444
P=0.501
P=0.873
P=0.068
Per IQR PM10 exposure
P=0.754
P=0.577
P=0.241
P=0.685
P=0.547
P=0.101
P=0.076
P=0.092
P=0.352
P=0.597
P=0.748
P=0.048
Per IQR PM2.5 exposure
P=0.423
P=0.534
P=0.467
P=0.493
P=0.340
P=0.277
P=0.069
P=0.285
P=0.575
P=0.899
P=0.723
P=0.242
Per IQR CO exposure
P=0.717
P=0.794
P=0.501
P=0.269
P=0.667
P=0.476
P=0.144
P=0.972
P=0.146
P=0.263
P=0.965
P=0.049
Per IQR SO2 exposure
P=0.726
P=0.040
P=0.875
P=0.618
P=0.541
P=0.096
P=0.999
P=0.344
P=0.731
P=0.045
P=0.846
P=0.010

Figure S4. Modification for the potential effects of air pollution exposure in association with GDM subgroups according to BMI in the first trimester. *P* value for interaction.

Abbreviations: GDM, gestational diabetes mellitus; GDM-IFH, GDM with isolated fasting hyperglycemia; GDM-IPH, GDM with isolated post-load hyperglycemia; GDM-CH, GDM with combined hyperglycemia; NO_2, nitrogen dioxide; O_3, ozone; PM_{10}, inhalable particulate matter; $PM_{2.5}$, fine particulate matter; CO, carbon monoxide; SO_2, sulfur dioxide; IQR, interquartile range.

Model adjusted for maternal age, gravidity, parity, tobacco, alcohol, folic acid, assisted reproduction, macrosomia, multiple pregnancies, the season of OGTT, temperature, and relative humidity.
